# Supplementary material for: Content-rich biological network constructed by mining PubMed abstracts
Source: BMC Bioinformatics. 2004 Oct 8;5:147. doi: 10.1186/1471-2105-5-147 (PMC528731; doi:10.1186/1471-2105-5-147)
Supplement: Additional File 2 — The original results of the above study (non-essential files are deleted to keep the file size under the limit set by BMC bioinformatics). [file 1471-2105-5-147-S2.bz2 › chilibotAdditionalFile2/dip05/739ID9393690E18116/html/OMPF_TOLB.html]

 


 **OMPF** and **TOLB** 
  
Found 6 abstracts in PubMed, retrieved 05.  
 

 What does Google say? 
 PDF only 
| .edu only 

---

**Interactive relationship** (e.g. stimulation, inhibition, etc)

**Neutral relationship**- **TolB**  interacted with the trimeric porins  **OmpF** , OmpC, PhoE, and LamB but not with their denatured monomeric forms or OmpA.  Ref: 9393690 J Bacteriol, 1997

**Non-interactive relationship** (e.g. studied together, co-existance, homology, etc.)

- In this study, we demonstrate that the genes tolQ, tolR, and tolA, but not  **tolB** , tolC, and  **ompF** , are required for the internalization of cloacin DF13 and they are not involved in the transport of ferric aerobactin.  Ref: 8419302 J Bacteriol, 1993
- Bacteriocin sensitivity tests performed on a collection of E. coli envelope mutants, and isolation and characterization of E. coli bacteriocin 28b insensitive mutants, showed that the core lipopolysaccharide, outer membrane proteins OmpA and  **OmpF** , and TolQ, TolA, and  **TolB**  proteins are involved in bacteriocin 28b lethal activity.  Ref: 8595593 Can J Microbiol, 1996
- We consider a variety of different colicins, including A, B, D, E1, E3, Ia, M and N, that penetrate through the porins  **OmpF** , FepA, BtuB, Cir and FhuA, to subsequently interact with a few targets in the periplasm, including TolA,  **TolB** , TolC and TonB.  Ref: 12423783 Biochimie< MedlineTA>Biochimie, 1996
